# Diagnostic strategy of metagenomic next-generation sequencing for gram negative bacteria in respiratory infections

**DOI:** 10.1186/s12941-024-00670-x

**Published:** 2024-02-01

**Authors:** Wenyan Liang, Qun Zhang, Qian Qian, Mingyue Wang, Yuchen Ding, Ji Zhou, Yi Zhu, Yu Jin, Xuesong Chen, Hui Kong, Wei Song, Xin Lu, Xiaodong Wu, Xiaoyong Xu, Shanling Dai, Wenkui Sun

**Affiliations:** 1 Department of Respirology and Critical Care Medicine, The First Affiliated Hospital of Nanjing Medical University, Nanjing, 210029 China; 2 Jiangsu Health Vocational College, Nanjing, 211800 China; 3 Department of Respiratory and Critical Care Medicine, The Affiliated Jiangning Hospital of Nanjing Medical University, Nanjing, China; 4 Department of Respiratory and Critical Care Medicine, Shanghai East Hospital, Tongji University, Shanghai, China; 5 Department of respiratory and critical care medicine, The Second Affiliated Hospital of Nanjing University of Chinese Medicine, Nanjing, Jiangsu 210000 China

**Keywords:** Metagenomic next-generation sequencing, Gram-negative bacteria, Interpretational approaches, Diagnostic accuracy

## Abstract

**Objective:**

This study aims to identify the most effective diagnostic method for distinguishing pathogenic and non-pathogenic Gram-negative bacteria (GNB) in suspected pneumonia cases using metagenomic next-generation sequencing (mNGS) on bronchoalveolar lavage fluid (BALF) samples.

**Methods:**

The effectiveness of mNGS was assessed on BALF samples collected from 583 patients, and the results were compared with those from microbiological culture and final clinical diagnosis. Three interpretational approaches were evaluated for diagnostic accuracy.

**Results:**

mNGS outperformed culture significantly. Among the interpretational approaches, Clinical Interpretation (CI) demonstrated the best diagnostic performance with a sensitivity of 87.3%, specificity of 100%, positive predictive value of 100%, and negative predictive value of 98.3%. CI’s specificity was significantly higher than Simple Interpretation (SI) at 37.9%. Additionally, CI excluded some microorganisms identified as putative pathogens by SI, including *Haemophilus parainfluenzae, Haemophilus parahaemolyticus*, and *Klebsiella aerogenes*.

**Conclusion:**

Proper interpretation of mNGS data is crucial for accurately diagnosing respiratory infections caused by GNB. CI is recommended for this purpose.

## Introduction

Lower respiratory tract infections (LRTIs) are the most common type of infectious disease and the leading infectious cause of mortality worldwide [1]. Early recognition and clearance of pathogens are crucial in the management of LRTIs. Microbial culture, a traditional method for bacterial recognition, suffers from low sensitivity and requires a time-consuming process. It yields negative results in approximately 40–60% of patients with acute infections and sepsis [2, 3]. The failure to recognize pathogens with traditional approaches may lead to extended hospitalizations, readmissions, and increased mortality and morbidity [4]. Clearly, conventional methods are no longer sufficient to meet current clinical needs.

A variety of molecular diagnostic techniques have been employed for the rapid and precise identification of pathogens. These techniques encompass Polymerase Chain Reaction (PCR) and metagenomic Next-Generation Sequencing (mNGS), which are utilized either as stand-alone methods or in conjunction with traditional culture-based approaches. mNGS offers a significant advancement over PCR in pathogen identification. Unlike PCR, which requires prior knowledge of the pathogen and is limited by potential false negatives, mNGS enables broad-spectrum, non-targeted detection of multiple pathogens simultaneously [5–8]. This method is not only more sensitive and faster but also provides comprehensive pathogen characterization [9]. Its ability to identify a wide range of pathogens, including low-abundance ones, without prior genomic information, makes mNGS particularly effective in complex diagnostic scenarios, surpassing the capabilities of PCR in both efficiency and scope. Many clinical reports demonstrate the diagnostic utility of mNGS in detecting pathogens, especially in respiratory tract infections [10, 11]. Despite widespread acceptance of mNGS in clinical settings, a standardized method for interpreting its results is lacking, complicating the interpretation of mNGS results.

The clinical applications of mNGS are more common in patients with high severity. Gram-negative bacteria (GNB) are critical pathogens and are increasingly significant in LRTIs for these patients [12]. Furthermore, GNB are the most commonly identified pathogens in mNGS studies [13–17]. However, the respiratory tract often experiences high levels of GNB colonization, and there is no widely accepted method for interpreting these results. Distinguishing between colonization and actual infection can be challenging for clinicians. Given this situation, we sought to study the distribution of Gram-negative bacteria in LRTIs using mNGS and evaluate different approaches for interpreting mNGS results.

## Materials and methods

### Study population and ethical considerations

Between June 1, 2020, and November 1, 2022, a total of 583 patients with suspected pulmonary infection were enrolled in this study, originating from four medical institutions located in China. All enrolled patients were required to meet the following criteria: Firstly, they had to present with symptoms such as fever, cough, expectoration, shortness of breath, dyspnea, and abnormal imaging findings. Secondly, bronchoalveolar lavage (BALF) samples were collected concurrently for both metagenomic next-generation sequencing (mNGS) and culture to identify pathogens. Thirdly, the quality inspection and BALF sample testing process met the standards of mNGS. Patients with confirmed Gram-positive bacterial infections, fungal infections, or infections caused by other pathogens were excluded from the analysis. Demographic and baseline characteristics, clinical presentation, radiography and laboratory findings, treatment, and outcomes of the 583 patients were investigated for clinical diagnosis. The study was approved by the Institutional Review Board of the First Affiliated Hospital of Nanjing Medical University (approval no. 2022-SR-014).

### Clinical groups of patients

Patients were stratified into three groups based on the presence of co-existing diseases. The Simple Pulmonary Infection Group: This group included patients without underlying diseases. The Immunosuppressed Group: This group comprised patients diagnosed with autoimmune diseases, post-splenectomy individuals, and/or those on long-term treatment with glucocorticoids, immunosuppressive agents, cytotoxic drugs, hematological malignancies, or those who had undergone chemotherapy in the last 6 months or solid organ transplantation. The Chronic Airway Disease Group: This group included patients with chronic bronchitis, bronchiectasis, or chronic obstructive pulmonary disease (COPD), but without immunosuppression.

### Specimen collection and processing

Bronchoalveolar lavage fluid (BALF) for metagenomic next-generation sequencing (mNGS) and traditional culture was collected by an experienced clinician using standard procedures from patients with suspected pulmonary infection. Before the procedure, patients’ nasal or oral cavities were cleansed with normal saline. Dexmedetomidine was administered for sedation before the bronchoscopy, and local anesthesia with 2% lidocaine was applied during the examination. The electronic bronchoscope was used to examine all bronchi in detail, and any lesions found on a chest CT scan were brush-examined before BALF collection. The area for lavage was determined based on the lesion area found on the chest CT scan, with BALF collected from the right middle lobe or the subsegment of the left lingual lobe if scattered lesions were present.

Approximately 100 mL of sterile normal saline was injected into the target bronchus in batches at 37 °C, with the first 20 mL discarded to avoid contamination, and approximately 5 mL collected into sterile tubes. The BALF samples were then divided into aliquots for pathogen detection, with one aliquot inactivated (56 °C, 30 min) before nucleic acid extraction.

### mNGS assay

(i) Nucleic Acid Extraction: Bronchoalveolar lavage fluid (BALF) samples were procured following standard protocols. DNA extraction employed the TIANamp Micro DNA Kit (Tiangen Biotech, Beijing, China), adhering to the manufacturer’s guidelines. Each batch included a no-template control (NTC) alongside clinical specimens. DNA quantification and quality assessment utilized Qubit and NanoDrop devices (Thermo Fisher Scientific). (ii) Library Preparation and Sequencing: The Hieff NGS C130P2 OnePot II DNA Library Prep Kit for MGI (Yeasen Biotechnology) facilitated DNA library construction, in line with manufacturer instructions. Post-preparation, libraries underwent Agilent 2100 qualification and were sequenced as 50 bp single-ends on DNBSEQ-200 (MGI Tech, China). Quality control (QC) criteria required over 18 ng of DNA post-library construction and a minimum of eight million raw reads. (iii) Bioinformatics Analysis: An in-house bioinformatics pipeline was employed for microorganism identification. Quality sequencing data were refined by removing low-quality reads, adapter contaminants, duplicates, and reads shorter than 36 bp. Human sequences were filtered out using bowtie2 software against the hs37d5 human reference genome. The residual data was aligned with the NCBI microorganism genome database using Kraken2, enabling the determination of the samples’ microbial composition.

### Culture method

The BLAF was inoculated onto bacteriological media, including blood agar, chocolate agar, and blue agar plates, using sterile wire loops. Incubation was carried out at 35 °C for 48 h in a 5% CO2 atmosphere within a thermostatic incubator. Dominant colonies were then selected for bacterial identification using the VITEK2-Compact, an automated system from BioMerieux, France. Bacterial strains identified in the BALF at concentrations of ≥ 10^3 colony-forming units per milliliter were deemed causative pathogens.

### Three interpretational approaches of mNGS

Simple Interpretation (SI): Gram-negative bacteria were identified by mNGS. Laboratory Interpretation (LI) [18, 19]: the parameter of gram-negative bacteria reached one of the following criteria: (i) relative abundance of pathogens detected by mNGS at the genus level was greater than or equal to 30%, regardless of the culture results, or (ii) the coverage rate scored 10-fold greater than that of any other microbes according to Langelier’s study. Laboratory interpretation serves as the positive standard for mNGS. Clinical Interpretation (CI): the bacteria identified in LI were further screened based on additional criteria. This microbe must have unambiguous literature evidence of its pulmonary pathogenicity, and the matched patient must have had risk factors for its infection.

### Clinical diagnosis

Two physicians with expertise in managing infections (WKS and XSC) conducted an independent review of all patient medical records, as well as the results of culture and mNGS. The physicians initially determined whether patients had an infectious or noninfectious etiology. Following this, they identified the causative pathogens by evaluating a combination of clinical manifestations, laboratory tests, chest radiology, and microbiological tests (including culture and mNGS). Any disagreements between the two intensivists were resolved through in-depth discussion, and another senior physician (SLD) was consulted if consensus could not be reached.

### Statistical analysis

Following the extracted data, 2 × 2 contingency tables were derived to determine sensitivity, and the McNemar test was used for discrete variables when appropriate. Differences between qualitative variables were assessed using the Fisher exact test, and the chi-square test was used to compare differences in positivity rates. Concordance was assessed using kappa statistics (kappa ≤ 0.4 low, kappa 0.41–0.6 fair, kappa > 0.6 good). Data analyses were performed using SPSS18 and GraphPad Prism7 software. P values smaller than 0.05 were considered statistically significant, and all tests were two-tailed.

## Results

### Patient characteristics

**Fig. 1:**
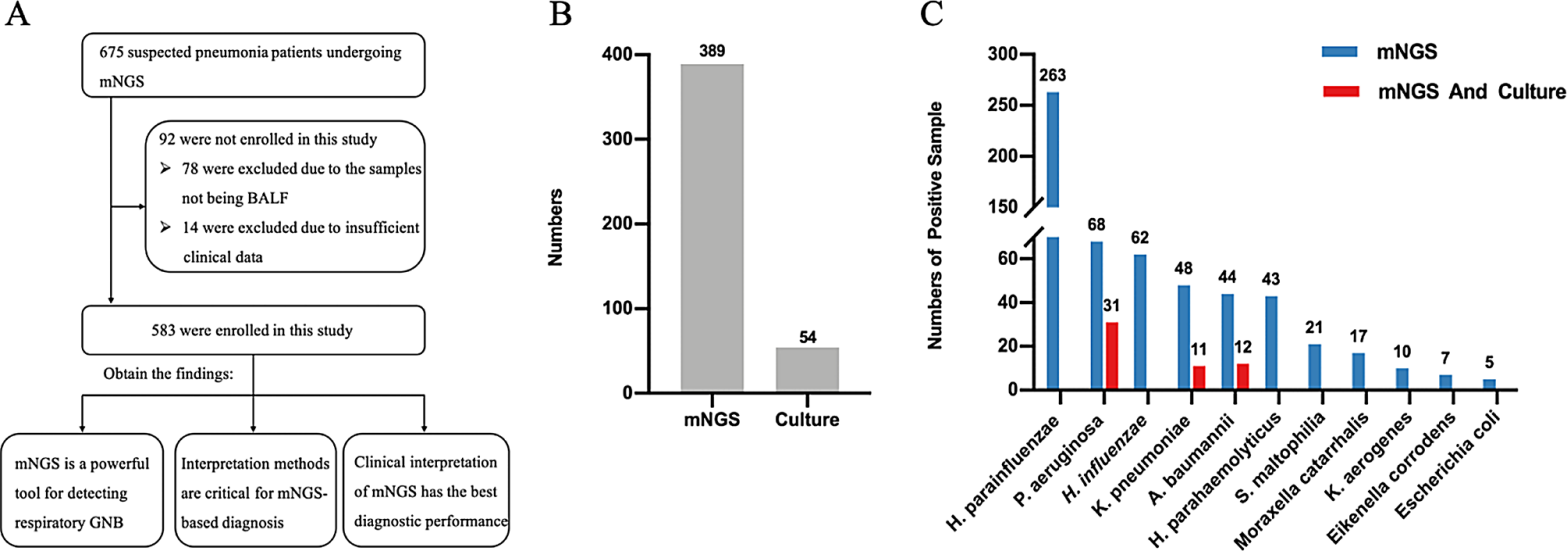
Detection performance of mNGS and culture. (**A**) The enrolled patients and research findings. (**B**) The comparison between mNGS and culture results in patients with positive findings. (**C**) The numbers and spectrum of bacteria detected with diffenent tools. *Abbreviations*: mNGS, metagenomic next-generation sequencing. *H. parainfluenzae, Haemophilus parainfluenzae. P. aeruginosa, Pseudomonas aeruginosa. H. influenzae, Haemophilus influenzae. K. pneumoniae, Klebsiella pneumoniae. A. baumannii, Acinetobacter baumannii. H. parahaemolyticus, Haemophilus parahaemolyticus. K. aerogenes, Klebsiella aerogenes. S. maltophilia, Stenotrophomonas maltophilia*

The study included a total of 583 patients (Fig. 1A), with a median age of 57 years (Table 1). Among these patients, 247 had at least one comorbidity. Chest CT scans revealed abnormalities in all patients, with solitary lesions being the most common radiologic finding upon admission (262, 45.4%). Bronchoscopy revealed abnormal secretions, mucosal abnormalities (such as erythema and edema), ulceration, and plague.

**Table 1:** Baseline characteristics of 583 patients included

Characteristics	Patients, n (%)
Age(years)	57
Male	312 (53.5)
Chronic respiratory disease	
Chronic obstructive pulmonary disease	33 (5.6)
Bronchiectasis	79 (13.5)
Other lung disease	47 (8.1)
Immunocompromised status	
Solid tumor	23(3.9)
Hematologic disorders	10 (1.7)
Immunosuppressive drugs	55 (9.4)
Radiographic changes of chest	
Solitary lesion	262 (45.40)
Multiple lesions	209 (35.58)
Diffuse lesions	112 (19.2)
Bronchoscopic appearance	
Hyperemia and edema	278 (47.85)
Ulceration and necrosis	50 (8.58)
Plaque	21 (3.68)
Abnormal secretions	228 (39.26)

### Comparison of gram-negative bacteria detection by mNGS and culture

Figure 1B illustrates the positive findings in both mNGS and culture. mNGS identified GNB in 389 patients (66.7%), while conventional culture-based methods detected GNB in only 54 patients (9.2%). Moreover, mNGS detected a broader spectrum of Gram-negative bacteria (11 types) compared to culture (3 types). The top five GNB identified by mNGS were *Haemophilus parainfluenzae* (*H. parainfluenzae*, 45.1%), *Pseudomonas aeruginosa* (*P. aeruginosa*, 11.6%), *Haemophilus influenzae* (*H. influenzae*, 10.6%), *Klebsiella pneumoniae* (*K. pneumoniae*, 8.2%), *Acinetobacter baumannii* (*A. baumannii*, 7.5%). In contrast, culture identified *P. aeruginosa* (5.3%), *K. pneumoniae* (1.8%), and *A. baumannii* (2.0%), all of which were also recognized by mNGS (Fig. 1C).

### Distribution of gram-negative bacteria across different groups

**Fig. 2:**
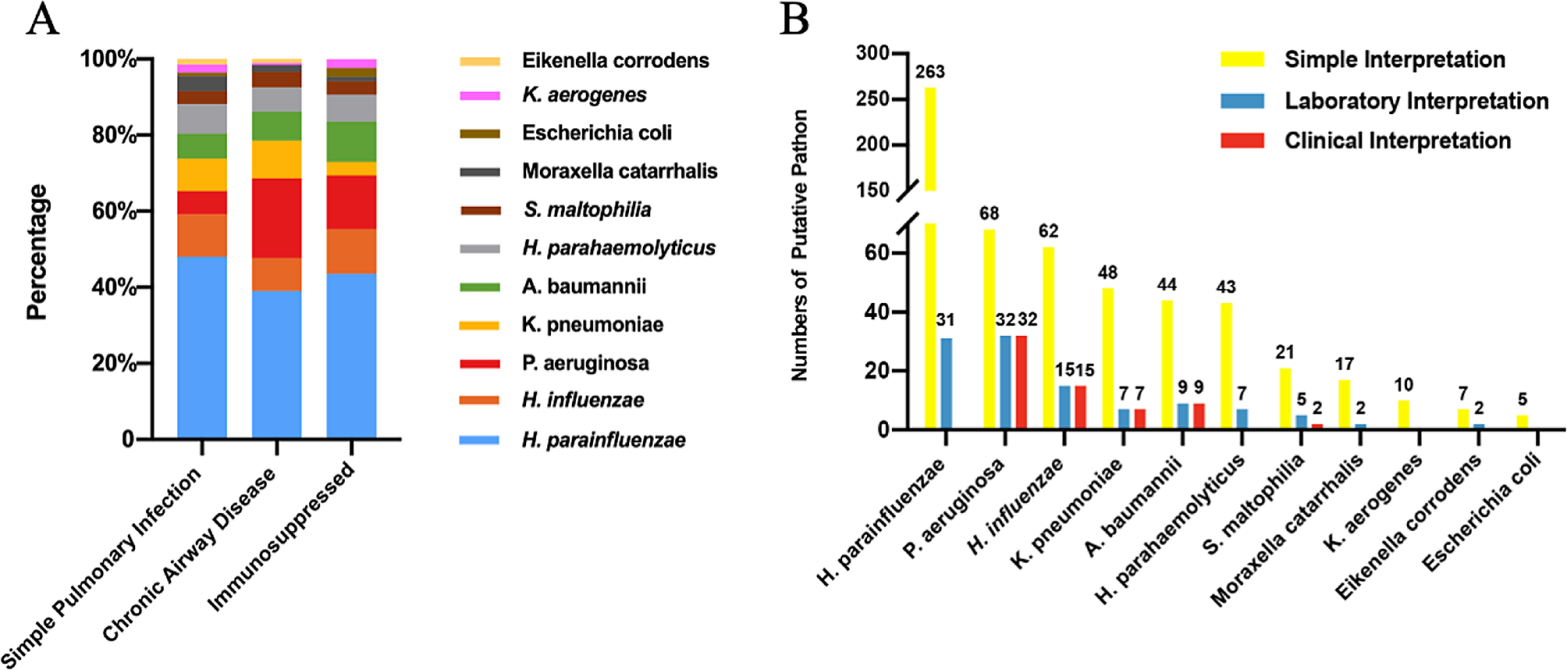
The distribution and identification of putative pathogen. (**A**) The distribution of identified bacteria in different clinical population. (**B**) The identification of putative pathogen in different interpretational approaches. *Abbreviations*: *h. parainfluenzae, Haemophilus parainfluenzae. P. aeruginosa, Pseudomonas aeruginosa. H. influenzae, Haemophilus influenzae. K. pneumoniae, Klebsiella pneumoniae. A. baumannii, Acinetobacter baumannii. H. parahaemolyticus, Haemophilus parahaemolyticus. K. aerogenes, Klebsiella aerogenes. S. maltophilia, Stenotrophomonas maltophilia*

We utilized mNGS to investigate the bacterial distribution among patients with varying underlying diseases and immune backgrounds. As depicted in Fig. 2A, H. *parainfluenzae* was the most frequently identified species across all groups, with the highest prevalence in the simple pulmonary infection group. Additionally, *P. aeruginosa* was more commonly found in the chronic airway disease group. No specific bacterial distribution was observed in the immunosuppressed group.

### Evaluation of putative pathogens via three interpretational approaches

Putative pathogens were analyzed using three distinct methods: simple Interpretation (SI), laboratory Interpretation (LI), and clinical Interpretation (CI). SI identified 588 pathogens, significantly more than LI and CI, which identified 110 and 65 pathogens respectively (Fig. 2B). Notably, bacterial species differed between SI and CI, with *H. parainfluenzae, Haemophilus parahaemolyticus* (*H. parahaemolyticus*), and *Klebsiella aerogenes* (*K. aerogenes*) excluded by CI.

### Diagnostic performance of MNGS across interpretational approaches

**Fig. 3:**
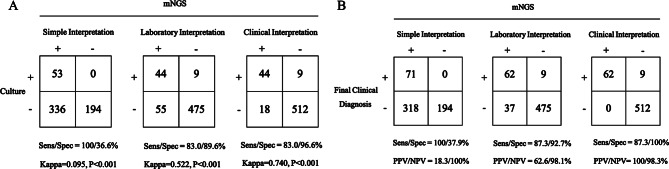
Diagnostic performance of mNGS with different interpretation. (**A**) 2 × 2 Contingency tables for culture. (**B**) 2 × 2 Contingency tables for final clinical diagnosis. *Abbreviations*: mNGS, metagenomic next-generation sequencing. Sens, sensitivity. Spec, specificity. NPV, negative predictive value. PPV, positive predictive value

A total of 389 (SI), 99 (LI), and 62 (CI) subjects were diagnosed with Gram-negative bacterial infections. Through comprehensive assessment by two clinical physicians, the final count of pulmonary GNB infection was 71. Sensitivity and specificity of mNGS testing compared to culture were as follows: SI, 100% (95% CI 91.5–100%) and 36.6% (95% CI 32.5–40.8%); LI, 83.0% (95% CI 69.7–91.4%) and 89.6% (95% CI 86.6–92.0%); CI, 83.0% (95% CI 69.7–91.4%) and 96.6% (95% CI 94.7–97.9%) (Fig. 3A). A consensus analysis revealed significant agreement (*p* < 0.001) but weak concordance (Kappa = 0.095) between SI and culture. When comparing to the final result by clinical physicians, the Positive Predictive Value (PPV) and Negative Predictive Value (NPV) were 18.3% (95% CI 14.6–22.5%) and 100% (95% CI 97.5–100%) for SI, 62.6% (95% CI 52.3–71.9%) and 98.1% (95% CI 96.4–99.1%) for LI, and 100% (95% CI 92.7–100%) and 98.3% (95% CI 96.6–99.2%) for CI (Fig. 3B). Among the approaches, CI demonstrated the highest PPV.

### Impact of antibiotic exposure on pathogen detection

**Fig. 4:**
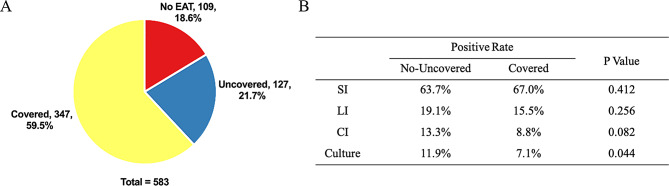
Impact of Antibiotic exposure on pathogen detection. (**A**) The pathogen coverage of EAT. (**B**) The effect of antibiotic exposure on detection rate for putative pathogens. *Abbreviations*: EAT, empirical antibiotic therapy. mNGS, metagenomic next-generation sequencing. SI, simple interpretation. LI, laboratory interpretation. CI, clinical interpretation. No-Uncovered, no eat and uncovered

In our study, 474 of 583 patients (81.3%) were administered antibiotics prior to mNGS and culture testing, while the remaining patients were not exposed to any antibiotic treatment. Empirical antibiotic regimens covered 59.5% of the gram-negative bacteria (Fig. 4A). The detection rate of putative pathogens by mNGS was analyzed between the Uncovered and Covered groups across three interpretational approaches (Fig. 4B). The detection rates in all three approaches were unaffected by antibiotic coverage. However, antibiotic coverage significantly reduced the culture detection rate to 7.1%, compared to 11.9% in the uncovered group (*p* = 0.044).

## Discussion

Lower respiratory tract infections are a significant source of morbidity and mortality in hospitalized patients [20]. GNB are considered canonical pathogens and are becoming increasingly important [21, 22]. In this study, we investigated the diagnostic accuracy of mNGS in detecting pulmonary infections caused by GNB. Our results demonstrated that mNGS was more effective than culture, but its effectiveness depended on the correct interpretation method.

In our study, mNGS identified a greater variety and number of GNB, including fastidious bacteria that are difficult to grow in culture. Fastidious GNB such as *H. influenzae, H. parainfluenzae, H. parahemolyticus, Eikenella corrodens*, and *Moraxella catarrhalis* are often found in the human respiratory tract and can cause disease under certain conditions [23, 24]. The presence of these bacteria in mNGS data underscores the need for careful analysis to avoid incorrect diagnoses. To address this, we established three different interpretive approaches.

The diagnostic accuracy of mNGS varied depending on the method used. While the sensitivity of mNGS testing was comparable to that of culture testing across all three approaches, SI had very low specificity and weak concordance with culture. In contrast, CI demonstrated the highest specificity and best concordance with culture. When compared to the confirmed cases, all three approaches had high Negative Predictive Values (NPV). The Positive Predictive Value (PPV) varied, with SI having the lowest at 18.3%, LI in line with other studies at 62.6%, and CI the highest at 100% [25, 26]^16^. This variation was attributed to the fact that BALF from the respiratory tract often mixes with oral flora and colonizers, leading to false-positive mNGS results [27]. Only CI effectively eliminated all false positives.

Some microorganisms may be excluded by CI. For example, *H. parainfluenzae* was ranked first in detection in our study, consistent with previous reports [28, 29], but was identified as a putative pathogen only by SI and LI, not CI. Similar situations occurred with other bacteria, such as *H. parahemolyticus* [30]. *Stenotrophomonas maltophilia (S. maltophilia)*, *Eikenella corrodens* and *Moraxella catarrhalis* were partially included by CI, reflecting their status as opportunistic pathogens that primarily infect specific populations with immunosuppression or structural lung disease [31–33]. Klebsiella aerogenes (*K. aerogenes*), an opportunistic pathogen often linked to severe infections in mechanically ventilated patients, was evaluated in this study [34–37]. The patient under investigation lacked pertinent risk factors and the bacterial sequence count fell short of our assessment criteria, leading to the exclusion of *K. aerogenes* from our considerations.

Core components in the WHO priority list [31, 38], such as *P. aeruginosa, K. pneumoniae, A. baumannii, H. influenzae*, and *Escherichia coli*, were identified as putative pathogens as long as they met the criterion of LI. These bacteria are responsible for a significant global clinical and epidemiological burden.

Unlike culture tests, mNGS is less affected by prior antibiotic usage, and it is widely accepted that antibiotics reduce the sensitivity of culture [13, 39]. In our study, the detection rate across all three approaches was unaffected by antibiotic coverage, although antibiotic coverage significantly decreased the detection rate in culture (11.9% vs. 7.1%, *p* = 0.044).

In our study, mNGS demonstrates efficacy in detecting respiratory GNB, markedly enhancing clinical pathogen diagnosis. Yet, the treatment of these pathogens encounters difficulties in drug selection, exacerbated by the rise of multifaceted drug-resistant strains. Our forthcoming research focuses on assessing the precision and dependability of mNGS in identifying resistance genes, thereby bolstering its theoretical foundation for clinical application in the detection of respiratory pathogens.

This study has limitations, including the need for a larger sample size, the lack of universally accepted criteria for mNGS, and the potential for subjective bias in the final diagnosis by clinical experts.

Overall, mNGS data require meticulous analysis to distinguish true pathogens from colonization. With proper interpretation, mNGS could become a valuable tool for precision diagnosis and tailored therapy for Gram-negative bacteria.

## Data Availability

The datasets used during the current study are available from the corresponding author on reasonable request.
